# Biobanking of COVID-19 specimens during the pandemic: The need for enhanced biosafety

**DOI:** 10.4102/ajlm.v10i1.1379

**Published:** 2021-10-21

**Authors:** Olayinka S. Ilesanmi, Aanuoluwapo A. Afolabi

**Affiliations:** 1 Department of Community Medicine, College of Medicine, University of Ibadan, Oyo State, Nigeria; 2 Department of Community Medicine, University College Hospital, Ibadan, Oyo State, Nigeria

Dear Editor,

Biobanking holds promising benefits particularly for improving the understanding of specific diseases and illnesses,^1^ as evidenced for the Zika virus disease. Research using biobanked blood samples helped resolve the ‘dengue-like syndrome’ misunderstanding associated with the Zika virus. Secondly, it provided comprehensive knowledge on the possibility for vertical transmission of the Zika virus between mother and child, as well as transmission via sexual relationships and blood transfusions.^2^ Biobanked biological samples could be kept for indefinite periods, allowing for long-term retrospective research in the future. The disease control opportunities that abound in biobanking can only be activated through efficient biobank structures.

Biobanks receive, process, store, and use available biological samples for research purposes to improve healthcare and reduce the incidence of disease occurrence. Biobanks are essential elements in the epidemiological surveillance of diseases and are often integrated into normal laboratory practices. Biobank staff are often trained on the rudiments of ethical guidelines for sample collection and handling and infection prevention and control measures.

The efficiency of biobanks is influenced by both internal and external factors. Internal factors could include the choice of specimen storage, information management, and communication technologies.^4^ External factors include ethical issues such as informed consent to use provided samples, conflict of interests of stakeholders (e.g. scientists, biobank administrators, sample donors, and commercial organisations), as well as commercialisation and profit distribution issues.

Inefficient handling and storage of specimens, information management systems, and communication modalities between sample handling and storage units of biobanks, as well as the commercialisation of samples, will reduce the efficiency of biobanks. This letter aimed to describe strategies for maintaining efficiency and safety in the coronavirus disease 2019 (COVID-19) biobanks.

Safety guidelines have been developed by the governments of many countries for COVID-19 biobanks.^5,6^ These guidelines mandate general laboratory practices and standard biosafety precautions but barely dwell on the sustenance of safety protocols.^7^ Laboratory safety could be maintained by regular training of biobank staff on COVID-19 guidelines and standard operating procedures, particularly specimen handling, risk assessments, emergency operating procedures, and overall safety precautions.^8^ This will ensure that laboratory managers and staff remain updated and maintain good laboratory practice. The use of personal protective equipment, such as disposable latex gloves, face shields, and laboratory gowns, is required for the safety of COVID-19 biobank staff.^8^ While preventing staff infection, personal protective equipment also reduces the risk of specimen contamination. Donning and doffing of personal protective equipment by biobank staff should be done in restricted areas, and proper handwashing when exiting the laboratory should be practised.

Individual COVID-19 samples must be handled as biohazardous material to prevent the infection of biobank staff. When COVID-19 samples are collected in designated laboratories, they are to be packaged and transferred to a biobank at 2 °C to –8 °C or frozen at –70 °C in a viral medium from the source laboratory^8^ and samples are to be transferred within 5 days of collection to a biobank. Affiliation with the nearest biobank is advised to allow for prompt transfer of collected COVID-19 samples.

Laboratory infrastructure may need to be reviewed to promote the efficiency of COVID-19 biobanks. The decontamination of specimen containers should be done before removing each specimen from the safety cabinet. In biosafety level-2 laboratories, routine testing should be automated and limited to inactivated specimens.^8^ Tasks involving the culture of severe acute respiratory syndrome coronavirus 2 must be carried out in biosafety level-3 laboratories.^9^ All examinations of COVID-19 specimens should be conducted in a certified Class II biological safety cabinet with a high-efficiency particulate air filter, which should be used alongside a high-efficiency particulate air-filtered incubator for culture samples obtained from suspected COVID-19 cases.^9^ Inactivated specimens should be stored in biosafety level-2 or higher safety laboratories and sample integrity must be ensured.

Periodic internal and external quality assurance assessment of laboratories handling COVID-19 biospecimens should be conducted to validate the generated results by biobanks. To achieve this, partnerships with public or private organisations with an excellent external quality assurance track record should be instituted. Internal quality assurance could be commenced and promoted through the establishment of a quality assurance unit within each biobank. Similarly, regular quality assurance training, reporting, and appraisal should be instituted.

The implementation of biosecurity measures should be implemented at all stages from sampling to labelling, tracking, and handling of COVID-19 specimens and results.^7^ Due to the highly infectious nature of COVID-19 samples, they could be targeted as weapons of bioterrorism, and biobank staff could be used to gain access to these samples. Thus, regulations such as a Material Transfer Agreement similar to those used in Middle East respiratory syndrome coronavirus biobanks should be developed. This will guard against the use of collected COVID-19 samples for personal, self-motivated purposes. Also, ethical considerations such as privacy, fairness, and beneficence must be considered when sharing COVID-19 specimen-related data among donors, researchers, and institutions.

Biobanked specimens could be used to conduct population-wide genetic sequencing research, which can identify severe acute respiratory syndrome coronavirus 2 mutation patterns among the population. Presently, COVID-19 vaccines such as Moderna, AstraZeneca, Pfizer, and Johnson & Johnson are being rolled out globally.^4^ However, many countries have reported that these vaccines are inadequate to cover the variants circulating within their population.^10^ Hence, prioritisation of certain populations may be needed during this period. Therefore, biobanking could provide essential information to influence the distribution of COVID-19 vaccines.

## Conclusion

Biobanks provide an opportunity through which insights can be gained into the diagnosis of diseases. They are also important for providing long-term storage of biological samples. While carrying out their roles, biobank staff could be at risk of COVID-19 infection. To prevent such occurrences, adherence to infection prevention and control measures should be promoted in biobanks. In addition, regular internal and external quality assurance should be ensured in all biobanks. To sustain biobanks, measures such as multisectoral collaboration are needed to ensure that the financial needs of biobanks are met.

## References

[1] Amin L, Hashim H, Mahadi Z, Ismail K. Determinants of the willingness to participate in biobanking among Malaysian stakeholders in the Klang Valley. MC Med Res Methodol. 2018;18:163. 10.1186/s12874-018-0619-2 PMC6282379 30518344

[2] Madhav N, Oppenheim B, Gallivan M, et al. Pandemics: Risks, impacts, and mitigation. In: Jamison DT, Gelband H, Horton S, et al., editors. Disease control priorities: Improving health and reducing poverty. 3rd ed. [homepage on the Internet]. Washington, DC: The International Bank for Reconstruction and Development/The World Bank; 2017. Chapter 17. Available from: https://www.ncbi.nlm.nih.gov/books/NBK525302/30212058

[3] Henderson GE, Cadigan RJ, Edwards TP, et al. Characterizing biobank organizations in the U.S.: Results from a national survey. Genome Med. 2013;5(1):3. 10.1186/gm407 23351549 PMC3706795

[4] Sargsyan K, Jaksa B, Hartl G, et al. Risk management in biobanks. In: J. Rocha, S. Oliveira 7 C. Capinha, editors, Risk Management and Assessment. London: IntechOpen. 10.5772/intechopen.91463

[5] Peeling RW, Boeras D, Wilder-Smith A, Sall A, Nkengasong J. Need for sustainable biobanking networks for COVID-19 and other diseases of epidemic potential. Lancet Infect Dis. 2020;20(10):E268–E273. 10.1016/S1473-3099(20)30461-8 32717208 PMC7380944

[6] Guidance COVID-19: A safe handling and processing for samples in laboratories [homepage on the Internet]. GOV.UK (Online). [cited 2020 Sept 09]. Available from: https://www.gov.uk/government/publications/wuhan-novel-coronavirus-guidance-for-clinical-diagnostic-laboratories/wuhan-novel-coronavirus-handling-and-processing-of-laboratory-specimens

[7] Cloudlims. COVID-19 clinical data management using LIMS [homepage on the Internet]. 2020. [cited 2020 Sept 10]. Available from: https://www.cloudlims.com/blog/covid-19-clinical-data-management-using-lims-html

[8] WHO. Laboratory testing for Coronavirus disease (COVID-19) in suspected human cases [homepage on the Internet]. World Health Organization; 2020. [cited 2020 Sept 09]. Available from: https://apps.who.int/iris/handle/10665/331501

[9] Yang J-R, Liu M-T, Huang H-I, Teng H-J, Chen J-H, Li S-Y. Building the national SARS-CoV-2 laboratory diagnostic capacity in Taiwan. Health Secur. 2020;18(5):383–391. 10.1089/hs.2020.0056 32822227

[10] Scroll. in. Coronavirus crises: Covid-19: Vaccine production and distribution has exposed – And intensified – Global inequality [homepage on the Internet]. [cited 2021 May 04]. Available from: https://scroll.in/article/992328/covid-19-vaccine-production-and-distribution-has-exposed-and-intensified-global-inequality

